# Supplementary material for: Global hypo-methylation in a proportion of glioblastoma enriched for an astrocytic signature is associated with increased invasion and altered immune landscape
Source: eLife. 2022 Nov 22;11:e77335. doi: 10.7554/eLife.77335 (PMC9681209; doi:10.7554/eLife.77335)
Supplement: Figure 2—source data 1. [file elife-77335-fig2-data1.zip › Figure_2_source_data_1/Figure_2B/homerResults/motif17.similar.html]

motif17

## Information for motif17

C
T
G
A
A
C
G
T
A
T
G
C
A
G
T
C
C
G
T
A
A
C
G
T
C
G
T
A
A
C
G
T
A
C
G
T
A
G
T
C
A
G
C
T
A
C
G
T
  
Reverse Opposite:  

C
G
T
A
C
T
G
A
A
C
T
G
G
T
C
A
C
G
T
A
A
C
G
T
G
T
C
A
A
C
G
T
C
T
A
G
T
A
C
G
C
G
T
A
A
G
C
T
  

|  |  |
| --- | --- |
| p-value: | 1e-59 |
| log p-value: | -1.361e+02 |
| Information Content per bp: | 1.902 |
| Number of Target Sequences with motif | 82.0 |
| Percentage of Target Sequences with motif | 3.55% |
| Number of Background Sequences with motif | 0.7 |
| Percentage of Background Sequences with motif | 0.21% |
| Average Position of motif in Targets | 90.7 +/- 52.3bp |
| Average Position of motif in Background | 44.0 +/- 0.0bp |
| Strand Bias (log2 ratio + to - strand density) | -0.0 |
| Multiplicity (# of sites on avg that occur together) | 1.03 |
| Motif File: | file (matrix) reverse opposite |

### Similar de novo motifs found

|  |  |  |  |  |  |  |  |
| --- | --- | --- | --- | --- | --- | --- | --- |
| Rank | Match Score | Redundant Motif | P-value | log P-value | % of Targets | % of Background | Motif file |
| 1 | 0.701 | C G T A A C T G C G T A C G T A A C G T A C G T A C G T A C T G C G T A C G T A | 1e-49 | -113.512696 | 3.16% | 0.38% | motif file (matrix) |
| 2 | 0.682 | C G T A C T A G C T A G C G A T C T G A C G A T T C G A C G A T | 1e-46 | -107.949881 | 6.23% | 1.46% | motif file (matrix) |
| 3 | 0.602 | C G T A A C G T C G T A C G T A A C G T C G T A A C G T C G T A A C G T C G T A A C G T A C G T | 1e-23 | -53.981940 | 2.77% | 0.68% | motif file (matrix) |
